# The Aerodynamic Cost of Head Morphology in Bats: Maybe Not as Bad as It Seems

**DOI:** 10.1371/journal.pone.0118545

**Published:** 2015-03-04

**Authors:** Dieter Vanderelst, Herbert Peremans, Norizham Abdul Razak, Edouard Verstraelen, Greg Dimitriadis

**Affiliations:** 1 School of Biological Sciences, Bristol University, Bristol, UK; 2 Department of Engineering Management, Active Perception Lab, University of Antwerp, Antwerp, Belgium; 3 School of Aerospace Engineering, Engineering Campus, Universiti Sains Malaysia, 14300 Nibong Tebal, Seberang Perai Selatan, Pulau Pinang, Malaysia; Università degli Studi di Napoli Federico II, ITALY

## Abstract

At first sight, echolocating bats face a difficult trade-off. As flying animals, they would benefit from a streamlined geometric shape to reduce aerodynamic drag and increase flight efficiency. However, as echolocating animals, their pinnae generate the acoustic cues necessary for navigation and foraging. Moreover, species emitting sound through their nostrils often feature elaborate noseleaves that help in focussing the emitted echolocation pulses. Both pinnae and noseleaves reduce the streamlined character of a bat’s morphology. It is generally assumed that by compromising the streamlined charactered of the geometry, the head morphology generates substantial drag, thereby reducing flight efficiency. In contrast, it has also been suggested that the pinnae of bats generate lift forces counteracting the detrimental effect of the increased drag. However, very little data exist on the aerodynamic properties of bat pinnae and noseleaves. In this work, the aerodynamic forces generated by the heads of seven species of bats, including noseleaved bats, are measured by testing detailed 3D models in a wind tunnel. Models of *Myotis daubentonii, Macrophyllum macrophyllum, Micronycteris microtis, Eptesicus fuscus, Rhinolophus formosae, Rhinolophus rouxi* and *Phyllostomus discolor* are tested. The results confirm that non-streamlined facial morphologies yield considerable drag forces but also generate substantial lift. The net effect is a slight increase in the lift-to-drag ratio. Therefore, there is no evidence of high aerodynamic costs associated with the morphology of bat heads.

## Introduction

Bats are the only flying animals with external pinnae, which in echolocating bats are often large with respect to their body size. For example, in the aerial interceptor *Eptesicus fuscus* the pinnae are 10–15% of the total body length and 70–80% in the gleaning bat *Plecotus auritus*. In addition to large pinnae, many bats vocalizing through their nose carry a prominent noseleaf [1]. For example, the gleaning bat [2] *M. microtis* has a noseleaf that is about 20% of its body length [3]. Therefore, at first sight, echolocating bats face an obvious trade-off. As flying animals, they would seem to benefit from a streamlined geometric shape to reduce drag and increase flight efficiency. However, as echolocating animals, their external pinnae generate the acoustic cues they rely on for navigation and hunting, e.g. [4–7]. In addition, in most bats vocalizing through their nostrils the streamlined design is further compromised by the noseleaf that helps in focussing the emission beam [3, 8–10].

It has been suggested that the pinnae and noseleaves of echolocating bats affect their aerodynamic efficiency, e.g. [11–15]. However, virtually no data on the aerodynamic properties of these structures are available. Using theoretical arguments, Bullen and McKenzie [16] and Gardiner et al. [15] highlighted the possible negative impact of pinnae on bat flight efficiency. Using a sample of 98 species, Gardiner et al. [15] confirmed that bats with slow hunting styles, so-called gleaning bats, tend to have larger pinnae than species with other hunting styles. This tendency was attributed to the drag created by large pinnae at higher flight speeds. In the only direct measurement of the aerodynamic forces generated by bat pinnae, Gardiner et al. [17] tested a simplified model of the bat *Plecotus auritus* (a gleaning bat without noseleaf). They found the pinnae to generate considerable drag forces as well as lift forces. However, the pinnae of *P. auritus* are extremely large (70–80%) with respect to its body size and it remains to be confirmed whether the results also hold for other bat species. In summary, the aerodynamic properties of pinnae and noseleaves remain largely untested and it is unclear whether these appendages constitute a net aerodynamic cost.

**Fig 1:**
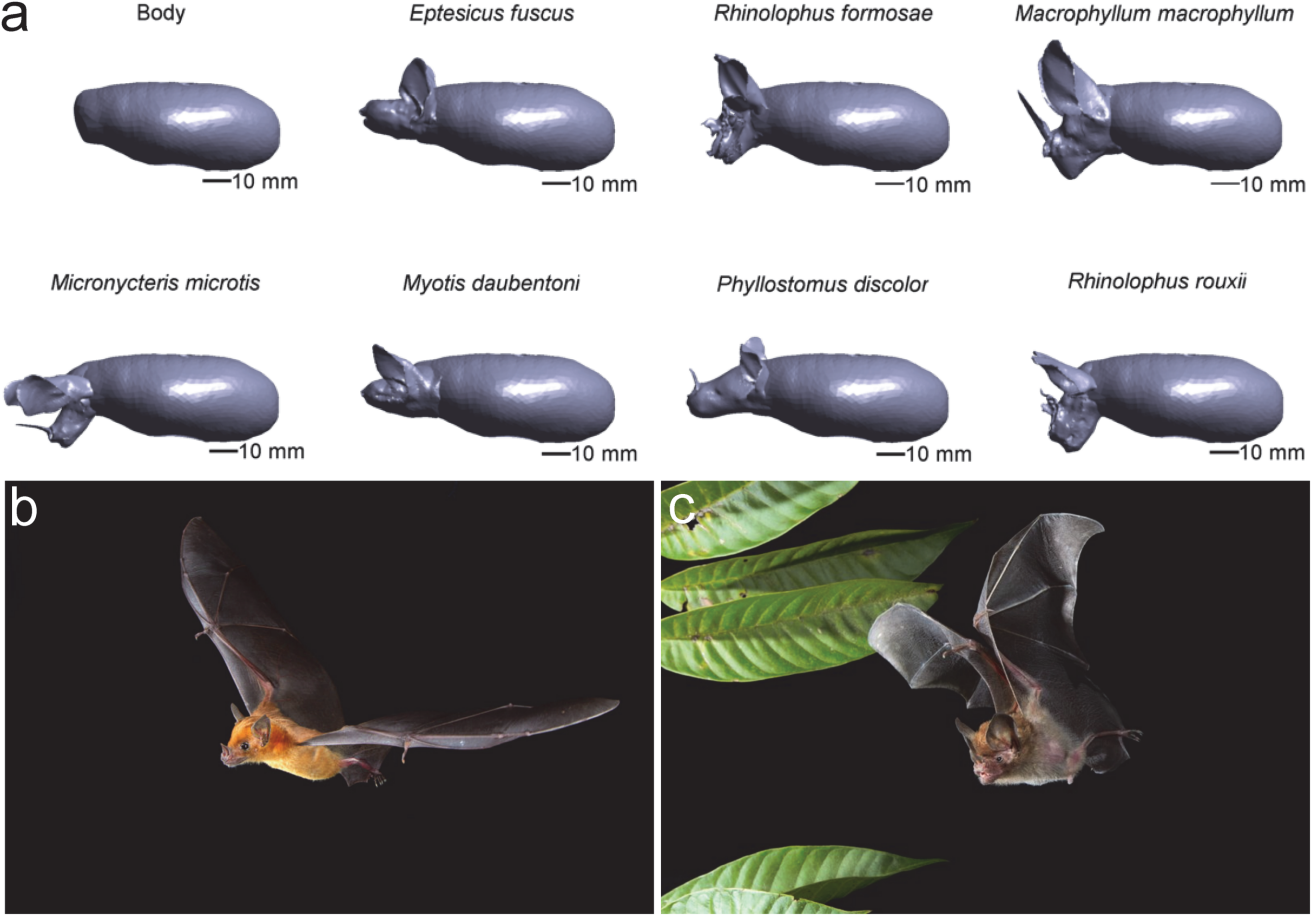
Bat models. (a) Renderings of the 3D bat models (orthographic projection). (b) Picture of *Phyllostomus discolor* in flight (c) Picture of *Micronycteris hirsuta* in flight. Both images copyright by Bruce D. Taubert and used with permission.

This paper quantifies the aerodynamic cost of the non-streamlined shape of bat heads, including species with noseleaves. To this end, the lift and drag forces acting on wind tunnel models of seven species of bat are measured. Lift is the upward force counteracting gravity. Drag is the force opposing forward motion exerted by the air on the bat. A detailed account of these terms can be found in reference [18]. Measuring the lift and drag forces generated by the models allows the calculation of the lift-to-drag ratio. This is a direct measure of aerodynamic efficiency [19, 20]. The aerodynamic forces were measured by testing detailed 3D printed models of the heads and bodies of the seven bat species in a wind tunnel (Fig. 1 and S1 supplementary material).

By testing a range of seven species, this paper extends the work of Gardiner et al. [15] to a wider range of species to include (non-gleaning) bats with moderate pinnae sizes. Moreover, by including species with noseleaves, we also quantify the cost of the head morphology in noseleaved bats. The seven species of bats tested in this paper are *Myotis daubentonii*, *Macrophyllum macrophyllum*, *Micronycteris microtis*, *Eptesicus fuscus*, *Rhinolophus formosae*, *Rhinolophus rouxi* and *Phyllostomus discolor*.

*M. macrophyllum* is a neotropical trawling bat. Relying on echolocation, it hunts over water taking insects directly from the water surface (trawling) or capturing them in the air just above the water (aerial hawking) [21]. This bat has a prominent noseleaf but, to the best of our knowledge, the acoustics of both noseleaf and pinnae remain unstudied. The hunting style of the European species *M. daubentonii* closely resembles that of *M. macrophyllum* [21, 22]. No data on the functionality of the pinnae have been published. *M. microtis* is a small insectivorous gleaning bat that preys on large insects in dense rainforest under story [2]. The acoustic effect of the noseleaf and pinnae of this bat has been studied by Vanderelst et al. [3]. The noseleaf and pinnae focus the emission beam and hearing allowing this bat to inspect a small portion of the world with each call. This strategy potentially improves the detection of prey sitting on leaves by reducing background clutter. *P. discolor* is an omnivorous bat that feeds on a wide range of food items including nectar, pollen, flowers, fruits and occasionally a few insects [23, 24]. The acoustic performance of both the pinnae [3, 25, 26] and the relatively small noseleaf [3] has been quantified. Comparing the directionality with that of *M. microtis*, Vanderelst et al. [3] found a lesser ability to focus in *P. discolor*. *R. formosae* and *R. rouxi* are constant frequency bats emitting long narrowband pulses [27, 28]. These species feature the baroque noseleaves typical for this genus. The acoustic function of the pinnae of *R. rouxi* has been investigated by [4, 29], while the focussing effect of the noseleaves of this genus has been the topic of a number of studies [9, 10, 30]. *E. fuscus* is an aerial interceptor hunting for insect prey on the wing [31]. The echolocation behaviour of this bat has been well documented. By filtering the echoes, the pinnae generate spectral cues that allow the E. fuscus to localize reflectors (i.e. flying prey) in azimuth and elevation [6, 32–35].

## Materials and Methods

A single head specimen was obtained for each species through loans from other labs on permits in accordance to the local law for each specimen. *M. daubentonii* was collected under the permit from the Danish Ministry of Environment, J.nr. NST-3446-00001 held by Prof. Annemarie Surlykke, Department of Biology, SDU, University of Southern Denmark, Odense Denmark. The specimen used for this project died of natural causes. The work with *M. microtis* and *M. macrophyllum* was approved by the Smithsonian Institutional Animal Care and Use Committee (IACUC; 2008-11-06-24-08) and the Panamanian Environmental Agency (ANAM, collecting permit number SE/A-78-08; export permit number SEX/A-100-08). *E. fuscus* died of natural causes while part of the animal colony at Maryland University and was collected using a permit from the Maryland Department of Natural Resources held by Prof. Cynthia F. Moss. *R. formosae* was mist-netted in Kenting in 2010 during a study supported by a National Science Council of Taiwan grant (99-2621-B-006-003-MY3) and a study permit (COA-TFRI-Heng-0990000188) to Prof. Ya-Fu Lee. The collection complied with the ethical guidelines and laws of the country. Both *R. rouxi* and *P. discolor* specimens were previously used in references [25, 29].

### Model generation

The aerodynamic forces were not measured directly on the head specimens. Instead, detailed 3D computer models were derived from micro-CT scans of the heads. Next, the models were physically realized by means of 3D printing. Finally, the 3D printed models were subjected to wind tunnel testing. Using models of the bat heads avoided decomposition of the specimens during lengthy wind tunnel tests. Also, as detailed below, using models allowed us to attach a standardized body to each of the heads. Below we describe the process used to generate the models for wind tunnel testing.

The single head for each species was scanned using a Skyscan 1076 micro-CT machine at a resolution of 35 *μm*. The scanning procedure and subsequent processing steps are detailed in reference [26]. In brief, the CT shadow images were reconstructed with the software provided by the manufacturer, resulting in a set of grayscale images for each bat. To decrease computer memory load, these data were downsampled to a resolution of 70 *μm*. The 3D voxel data were segmented by separating the tissue from the background using a biomedical imaging software package (Amira, USA). The separation process was carried out automatically as much as possible, with some manual corrections. Initial mesh models were rendered from the segmented data; they were smoothed and re-meshed several times in order to decrease the number of triangles. More details about the construction of the 3D bat models can be found in references [3–5, 9, 10, 26, 36].

Measuring the aerodynamic properties of the isolated heads was deemed invalid. Such a procedure would create trailing wakes behind the heads [15] that would artificially increase the drag. In real bats, the space behind the head is occupied by the body and such a wake would not develop. Hence, a standardized body was created using 3D modeling software (EasyToy, Livesforce, China). The shape of the body was modeled after profile photos of flying bats. The body model was scaled to a length of 72 mm. It was assumed that the heads of bats measure between 20% and 30% of the total body length. For example, for *E. fuscus*, *M. macrophyllum*, *Rhinolophus paradoxolophus* and *P. discolor* and the head to body length ratio is about 20% [37], 25% [38], 25% [39] and 30% [23] respectively. The models’ heads were scaled up or down accordingly, the scaling factors ranging from 0.9 (*P. discolor*) to 2.0 (*M. macrophyllum*). Hence, the heads were scaled by much less than an order of magnitude, ensuring that the flow characteristics were not altered significantly by the scaling operation [17]. When merging the head models with the body model, some overlap was necessary to ensure a smooth transition between the two parts. Consequently, the final head to body length ratio of the bat models ranged from 25% to 30%, which is in close agreement with the ratios mentioned above.

During flight, bats are able to use head movements to direct their echolocation beam to locations of interest [40]. The complete range of head orientations that can be adopted by the seven species cannot be represented by static models. Instead, it was decided to model a representative head posture for each species. The orientation of six of the seven heads approximated the posture of the head during flight as determined from in-flight pictures for each species (See Fig. 1 for an example for *P. discolor* and supporting material for more in-flight photographs). The head of the seventh species, *M. microtis*, was oriented based on its simulated emission directionality (see below).

In-flight images suggest that *P. discolor*, *E. fuscus* and *M. daubentonii* keep their heads more or less level. For the two Rhinolophidae, the head position was again approximated from images. Moreover, the modeled head postures also resulted in the simulated emission beam to be centred just below zero degrees in elevation [4, 5, 9] as is typically found in other bat species [41]. Therefore, the head postures for *R. rouxi* and *R. formosae* could be taken to represent those of bats looking straight ahead. Likewise, the head posture of *M. macrophyllum*, a bat that hunts insects by trawling the water surface, was modelled after the image linked in the supplementary material.

High quality in-flight images for *M. microtis* could not be found. However, in-flight photos of *Micronycteris hirsuta* (Fig. 1), a species closely related to *M. microtis*, suggest that it holds its head in a horizontal position, akin to the posture modelled for *P. discolor*, *E. fuscus* and *M. daubentonii*. On the other hand, acoustic simulations show that for *M. microtis* this head posture would result in a beam that points upwards [3], which contrasts with the known beam directionality of other species [41]. We conjecture that the more horizontal head posture might be assumed whenever *M. microtis* hunts for silent prey on leaves using the so-called mirror effect ([2], also see http://www.youtube.com/watch?v=wXuGlIp07mc). Acoustic considerations suggest that *M. microtis* should tilt its head forward to obtain the same field of view as most other bats [3, 41]. Consequently, the model’s head was oriented in a more tilted position, such that the direction of the highest sensitivity of the simulated emission beam [3] corresponded to the direction of most other bat species for which the emission beam is known [3, 41]. Therefore, the maximum sensitivity of the simulated beam pattern for *M. microtis* was located at around −10 degrees in elevation [3].

The final models (Fig. 1) were produced by means of 3D Polyjet printing (Easy2day, Odense, Denmark) providing fittings for a 6-component force and torque balance (Supporting material). The lift and drag forces of all 7 species were measured in a wind tunnel for flight speeds of 5 and 10 ms. There is very little data about the flight speeds of the included species under natural conditions. Holderied [42] (partly republished in reference [43]) reports on the flight speed of a number of bat species and relates this to their mass. Based on this data, the 5 and 10 ms conditions cover the lower and upper flight speed range of bat species with a weight from about 8 to 40 g. To isolate the effect of the head morphology, the standardized body model without a head was also tested and used as a reference.

### Wind tunnel experiments

Each of the seven bat models and the standardized body model were tested in the wind tunnel facility of the University of Liège, Belgium. The wind tunnel is of Göttingen type [44] with two working sections. The tests were performed in the aeronautical section of the wind tunnel facility, which measures 2 m × 1.5 m × 5 m (W × H × L). The airspeed range is 2–60 ms with speed non-uniformity lower than 0.5% and a turbulence level of 0.15%. The wind speed was measured by a Pitot tube connected to a pressure transducer. The wind speed measurement is accurate to 0.1 ms.

The aerodynamic forces acting on the model were measured at several values of the angle of attack using the Nano17 6-axis Force/Torque Transducer by ATI Industrial Automation, NC, USA. The accuracy of the Nano17 is 1/320 N in force and 1/64 Nmm in torque.

A custom-made support system was designed and built in order to support the bat models in the wind tunnel and to allow accurate and efficient control of the angle of attack (Supporting material). This control is carried out by means of a stepper motor connected to a 3D-printed gearbox. The gearbox rotates the bat model support axis, the angle being measured by a potentiometer. A logic board was built to control the stepper motor, measure the pitch angle and communicate wirelessly with a computer outside the wind tunnel’s working section. The angles of attack were set to within 0.1° and verified using a digital inclinometer.

Models were tested for angles of attack between −30° and +30° in steps of 2.5°. Each combination of model and angle of attack was tested at two airspeeds to investigate the effect of the Reynolds number on the non-dimensional force coefficients. Therefore, for each bat model and pitch angle, three recordings were taken, at 0 ms, 5 ms and 10 ms respectively. Each recording consisted of 5000 samples of all the six forces and moments, taken over 10 seconds.

The force and moment values measured were very low (especially at 0 ms and 5 ms) but compatible with the measurement sensitivity of the sensor. The mean values of the recordings at 0 ms were subtracted from those obtained at wind-on conditions to remove gravity effects from the data. The resulting mean values were converted to non-dimensional drag coefficients and lift coefficients. The data were smoothed using a robust discretized smoothing spline [45, 46].

## Results

**Fig 2:**
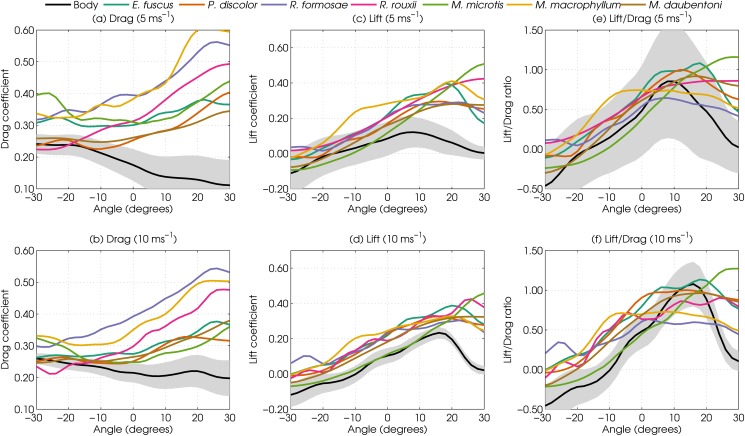
Aerodynamic test results. a-b; The drag coefficients for the 7 species of bats as a function of tilt angle at 5 and 10 ms. Negative tilt angles indicate that the complete bat model was tilted nose-down, c-d; The lift coefficients as a function of tilt at 5 and 10 ms for each model. e-f; The lift-to-drag ratio as a function of tilt at 5 and 10 ms. The shaded area denotes 1 standard deviation. The lift (drag) coefficient is calculated by multiplying the lift (drag) force by $m=\frac{1}{q\cdot A}$. With *q* the dynamic pressure and *A* the approximate surface area of the standardized body model. *m* is a constant for all conversions at the same airspeed.

**Fig 3:**
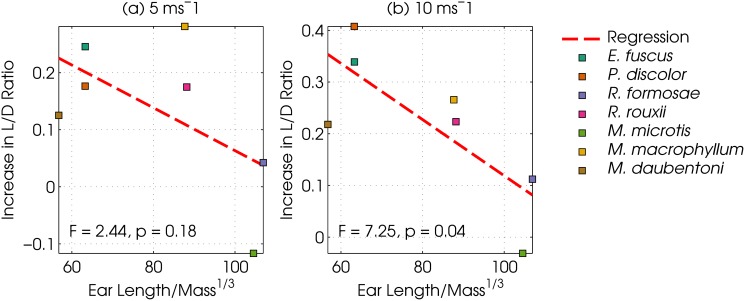
Flight cost as a function of normalized pinna length. Increase in lift to drag ratio due to the pinnae measured in the 7 models as a function of normalized pinna length, averaged across the full range of angles of attack. The normalized pinna length is calculated by dividing the pinna length by the cube root of the mass of the bat. Panel a: data for the 5 ms condition. Panel b: data for the 10 ms condition. The lines are least squares linear regression lines. The significance test results are indicated in both panels. Colours are the same as in Fig. 2

The drag for all bat models at virtually all angles of attack is higher than for the standardized body model—both at 5 ms and 10 ms (Fig. 2). For example, at angles of attack around 0°, the drag coefficient of the bat models is up to 100% higher than that of the standardised model (*R. formosae*). The heads also generate lift, resulting in lift coefficients higher than that of the standardised body for all species and angles of attack at both speeds. In absolute terms, the lift forces are considerable. Across all tested angles of attack and species, the lift force ranges from about 1 to 5 percent of the bat weight in the 5 ms condition. However, in the 10 ms condition, the lift ranges from 2 to 16 percent of the body weight (Table 1). The lift generated by the heads resulted in a small net increase in the lift-to-drag-ratio for all species for sub-ranges of the tested angles of attack. Averaged across the range of angles of attack investigated, all species show a small increase in lift to drag ratio, except for *M. microtis* (Fig. 3).

**Table 1:** Lift forces compared to body weight. The lift forces listed are the differences in lift between each bat model and the body model averaged across tilt angles between −10 and 10 degrees. The lift forces have been adjusted for the differences in scaling of the tested bat models. Data on the weight of each species were taken from the literature.

Species	Mass, *g*	Weight, *mN*	Lift, *mN*		% of Weight	
			5 $\frac{m}{s}$	10 $\frac{m}{s}$	5 $\frac{m}{s}$	10 $\frac{m}{s}$
E. fuscus	20	196.1	5.4	20.3	2.8	10.4
P. discolor	42	411.9	6.6	40.6	1.6	9.9
R. formosae	21	205.9	10.2	33.3	5.0	16.2
R. rouxii	10	98.1	3.5	13.6	3.6	13.8
M. microtis	6	58.8	0.7	1.0	1.2	1.7
M. macrophyllum	8	78.5	3.4	10.2	4.3	13.0
M. daubentoni	10	98.1	1.5	6.8	1.5	6.9

**Fig 4:**
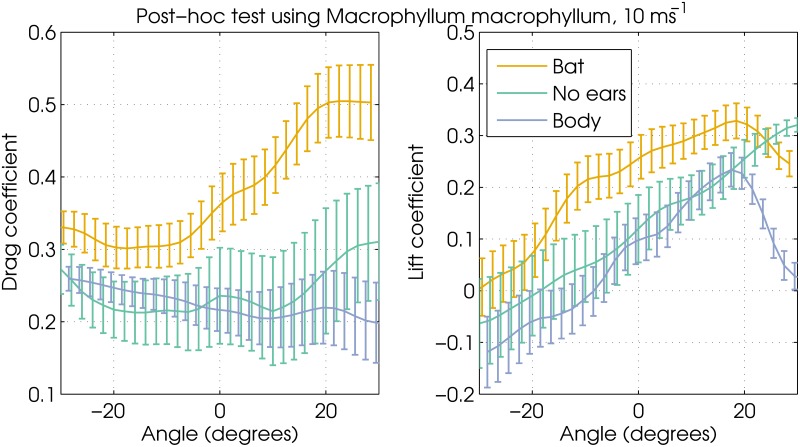
Left: Drag coefficients for two version of the *Macrophyllum macrophyllum* model and the standardized body model. Bat: the original model, No pinnae: pinnae removed from the original model, Body: the standardized body model. Right: idem for the lift coefficient. Vertical bars denote 1 standard deviation.

The pinnae are the only structures common to all seven species that could conceivably create substantial lift and drag [13, 15]. To verify that the increase in lift and drag forces can be attributed predominantly to the pinnae, we conducted a post-hoc measurement using the *M. macrophyllum* model (Fig. 4). Fig. 2 shows that this species, which features both noseleaf and pinnae, experiences high lift and drag forces at both windspeed settings. Therefore, this species was considered an appropriate model for post-hoc testing. The model was re-tested in the wind tunnel after subsequently removing the pinnae and the noseleaf. The results confirm that the aerodynamic difference between the bat model and the standardized body model are due to the pinnae, since the aerodynamic forces on the model without pinnae are very similar to those measured on the standardized body model (Fig. 4).

## Discussion

Birds do not rely on pinnae to localize sound [47]. They typically have streamlined head profiles that strongly contrast with the appendages featured by echolocating bats, such as large pinnae and noseleaves. This raises the question whether this departure from a streamlined design comes at a considerable aerodynamic cost. In light of the limited data available (exception: reference [17]) on the aerodynamics of bat heads we set out to measure the aerodynamic forces generated by the heads of seven species.

We found the heads of seven bat species to substantially increase drag by up to 100%. In addition, the head models also generate substantial lift. At 10 ms the lift ranged between 16% and 60% of the body weight of the bats. This lift effectively offsets the detrimental drag generated by the head morphology resulting in a slight net increase in lift-to-drag ratio for all bats, except for *M. microtis*. However, even for this species, there was no net *decrease* in lift to drag ratio. The finding that *M. microtis* is the only species without net increase in lift to drag ratio could be explained either by the fact that its head was oriented somewhat differently than the heads of the other species (see Methods) or by it having the largest pinnae relatively to its body size (about 50% [48]). That said, overall our results highlight the possibility that the aerodynamic effect of the non-streamlined head morphology of bats is not as detrimental as it might seem at first sight. Based on our data, the head morphology (including pinnae and noseleaf) does not necessarily constitute a trade-off, as is currently supposed [11, 12, 14, 15], in the sense that aerodynamic efficiency has been sacrificed to allow for better acoustic performance. If this had been the case, then the lift-to-drag ratio of the bat models would be expected to be lower than that of the standardized body model.

The only structures shared by all bats that conceivably generate the lift and drag forces are the pinnae. In a post-hoc test using *M. macrophyllum* we confirmed that the pinnae are responsible for creating both lift and drag. Our study is not the first to suggest that bat pinnae might generate substantial lift; to the best of our knowledge, the possibility that bat pinnae generate lift was first suggested in 1959 by Vaughan [13] in a study on the behaviour of *Eumops perotis*, a bat with particularly large pinnae. He suggested that lift generated by the pinnae might help the bats in keeping their heads up during flight. In the only experimental assessment of the aerodynamics of bat pinnae, Gardiner et al. [17] used a simplified physical model of *Plecotus Auritus* to confirm that pinnae have the potential to generate lift as well as drag. Our study extends the findings of Gardiner et al. [17] by testing more detailed models of a range of species, including species with moderate pinnae lengths (e.g. *E. fuscus*). In addition, we also tested species featuring a noseleaf—another feature compromising the streamlining of bat geometry.

The large variability in head shapes among bats is well documented [49] and the heads might not have a beneficial lift-to-drag ratio in all species. However, the present study concerns bats from very different ecological niches (two water gleaners, two aerial hawkers, a surface gleaner and two perch hunters), which suggests that the findings could be generalized across bat taxa. In extreme cases, however, the head morphology might represent an aerodynamic cost. The present data shows that the increase in lift-to-drag ratio is smaller for bat species with larger pinnae (Fig. 3a-b), suggesting that the very large pinnae of some gleaning bats might come at a net aerodynamic cost. Therefore, the heads of specialized gleaners such as *Plecotus spp*. or *Megaderma spp*., should be tested separately for their aerodynamic properties [17]. However, deriving 3D models for these species using currently established method has proven to be difficult as their pinnae tend to deform during the CT scanning process.

Using 3D printed, static models unavoidably introduces a number of limitations. For example, it is apparent that at least the large eared species will have some interaction between the ear wake and the inner wing or body junction and therefore modify the overall lift to drag ratio. However, modelling a realistic flapping bat wing was beyond the scope of this study. Furthermore, It would be incorrect to add a static wing or wing stub on the bat models as the bat species studied here do not glide they always flap. The interference effect of pinnae on a static wing cannot represent the true interference effect on a flapping wing. A second limitation of the current study is the focus on fixed head postures. Bats are known to vary their head posture during flight to steer their emission beam. However, these movements are not well documented and data are available for a limited number of species only [40, 50]. Hence, it was decided to measure the forces for a relevant fixed head posture. Be that as it may, once properly documented, assessing the effects of alternative in-flight head postures would be a logical extension of the current work.

The limitations associated with testing aerodynamics properties using static models makes corroborating evidence from experimental studies necessary before conclusive statements can be made. Still, our data highlight the possibility that instead of decreasing flight efficiency, the non-streamlined character of bat pinnae is virtually neutral in this respect. In conclusion, we suggest that pinnae and nose leaves do not constitute a considerable aerodynamic cost and call for a direct experimental assessment of the aerodynamic properties of bat morphologies.

## Supporting Information

S1 Links to online images of bats and pictures of the windtunnel setup. (PDF)
