# Correction: The Aerodynamic Cost of Head Morphology in Bats: Maybe Not as Bad as It Seems

**DOI:** 10.1371/journal.pone.0126061

**Published:** 2015-05-12

**Authors:** Dieter Vanderelst, Herbert Peremans, Norizham Abdul Razak, Edouard Verstraelen, Grigorios Dimitriadis

The fifth author’s name is spelled incorrectly. The correct name is: Grigorios Dimitriadis.

Additionally, there are errors in the author affiliations. The affiliations should appear as shown here:

Dieter Vanderelst^1,2,^ Herbert Peremans^2^, Norizham Abdul Razak^3^, Edouard Verstraelen^4^, Greg Dimitriadis^4^

1 School of Biological Sciences, Bristol University, Bristol, UK, 2 Department of Engineering Management, Active Perception Lab, University of Antwerp, Antwerp, Belgium, 3 School of Aerospace Engineering, Engineering Campus, Universiti Sains Malaysia, 14300 Nibong Tebal, Seberang Perai Selatan, Pulau Pinang, Malaysia, 4 Aerospace and Mechanical Engineering Department, University of Liège, Liège, Belgium.

## References

[1] Vanderelst D, Peremans H, Razak NA, Verstraelen E, Dimitriadis G (2015) The Aerodynamic Cost of Head Morphology in Bats: Maybe Not as Bad as It Seems. PLoS ONE 10(3): e0118545 doi: 10.1371/journal.pone.0118545 25739038 10.1371/journal.pone.0118545 PMC4349651

