# Supplementary material for: The Aerodynamic Cost of Head Morphology in Bats: Maybe Not as Bad as It Seems
Source: PLoS One. 2015 Mar 4;10(3):e0118545. doi: 10.1371/journal.pone.0118545 (PMC4349651; doi:10.1371/journal.pone.0118545)
Supplement: S1 — (PDF) [file pone.0118545.s001.pdf]

The aerodynamic costs of head morphology in bats

Supporting Material

Images of bats in flight

Below, we provide a number of links to online photographs of the species included in the paper. If no suitable pictures were found, a link to a closely related species is provided instead.

Myotis daubentonii

- *http://www.arkive.org/daubentons-bat/myotis-daubentonii/image-A22873.html*
- *http://www.arkive.org/daubentons-bat/myotis-daubentonii/image-A7188.html*

Rhinolophus rouxi and Rhinolophus formosae

- *http://www.arkive.org/lesser-horseshoe-bat/rhinolophus-hipposideros/image-A4165.html*

Eptesicus fuscus

- *http://www.arkive.org/big-brown-bat/eptesicus-fuscus/image-G142494.html*
- *http://dwrcdc.nr.utah.gov/rsgis2/search/Display.asp?FlNm=eptefusc*

Phyllostomus discolor

- *http://www.pbase.com/brucetaubert/image/141227100*

Micronycteris microtis

- *http://www.pbase.com/brucetaubert/image/141227123*

Macrophyllum macrophyllum

- *http://www.animalsandearth.com/en/photo/view/id/315523-long-legged-bat-macrophyllum-macrophyllum-flying-over-water-surface-looking-for-dead-and-drowning-insects-smithsonian-tropical-research-station-barro-colorado-island-panama*

Wind tunnel setup

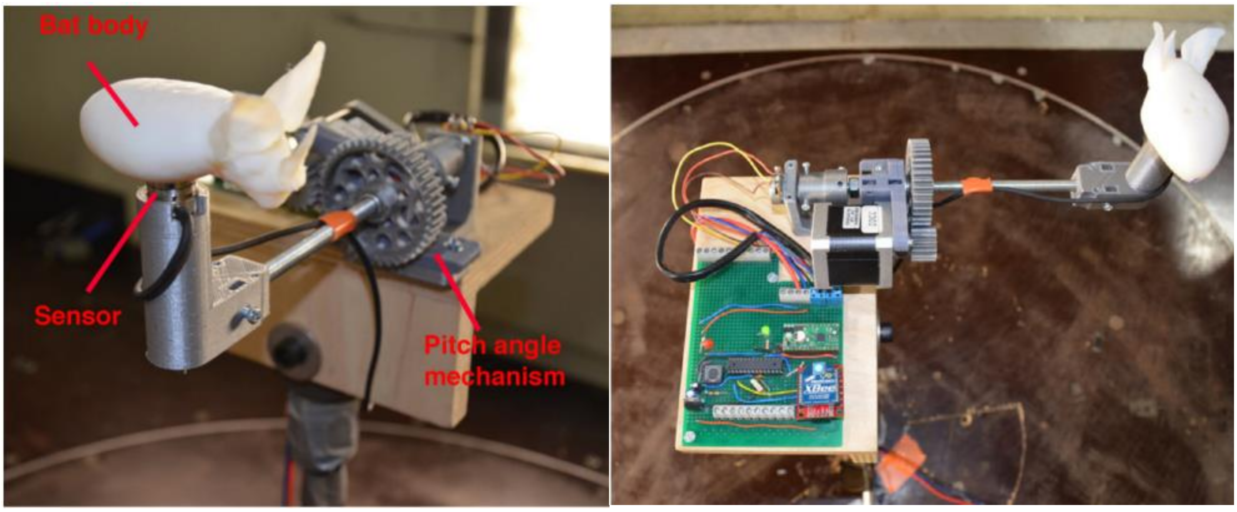

Photos depicting the bat model support system used for the wind tunnel tests. (Left) side view; (Right) top view.
